# Dynamic contrast-enhanced MRI in malignant pleural mesothelioma: prediction of outcome based on DCE-MRI measurements in patients undergoing cytotoxic chemotherapy

**DOI:** 10.1186/s12885-022-09277-x

**Published:** 2022-02-20

**Authors:** Martina Vivoda Tomšič, Peter Korošec, Viljem Kovač, Sotirios Bisdas, Katarina Šurlan Popovič

**Affiliations:** 1 University Clinic of Pulmonary and Allergic Diseases Golnik, Golnik 36, 4204 Golnik, Slovenia; 2 Faculty of Medicine, University of Ljubljana, Korytkova ulica 2, 1000 Ljubljana, Slovenia; 3 Faculty of Pharmacy, University of Ljubljana, Aškerčeva cesta 7, 1000 Ljubljana, Slovenia; 4 Department of Radiotherapy, Institute of Oncology Ljubljana, Zaloška cesta 2, 1000 Ljubljana, Slovenia; 5 Lysholm Department of Neuroradiology, National Hospital of Neurology and Neurosurgery, UCLH, London, UK; 6 Institute of Radiology, University Medical Centre Ljubljana, 1000 Ljubljana, Slovenia

**Keywords:** Mesothelioma diagnostic imaging, Mesothelioma drug therapy, Magnetic resonance imaging, Perfusion, Prognosis, Cisplatin, Survival, Progression free survival

## Abstract

**Background:**

The malignant pleural mesothelioma (MPM) response rate to chemotherapy is low. The identification of imaging biomarkers that could help guide the most effective therapy approach for individual patients is highly desirable. Our aim was to investigate the dynamic contrast-enhanced (DCE) MR parameters as predictors for progression-free (PFS) and overall survival (OS) in patients with MPM treated with cisplatin-based chemotherapy.

**Methods:**

Thirty-two consecutive patients with MPM were enrolled in this prospective study. Pretreatment and intratreatment DCE-MRI were scheduled in each patient. The DCE parameters were analyzed using the extended Tofts (ET) and the adiabatic approximation tissue homogeneity (AATH) model. Comparison analysis, logistic regression and ROC analysis were used to identify the predictors for the patient’s outcome.

**Results:**

Patients with higher pretreatment ET and AATH-calculated *K*^trans^ and v_e_ values had longer OS (*P*≤.006). Patients with a more prominent reduction in ET-calculated *K*^trans^ and k_ep_ values during the early phase of chemotherapy had longer PFS (*P* =.008). No parameter was identified to predict PFS. Pre-treatment ET-calculated *K*^trans^ was found to be an independent predictive marker for longer OS (*P*=.02) demonstrating the most favourable discrimination performance compared to other DCE parameters with an estimated sensitivity of 89% and specificity of 78% (AUC 0.9, 95% CI 0.74-0.98, cut off > 0.08 min^-1^).

**Conclusions:**

In the present study, higher pre-treatment ET-calculated *K*^trans^ values were associated with longer OS. The results suggest that DCE-MRI might provide additional information for identifying MPM patients that may respond to chemotherapy.

**Supplementary Information:**

The online version contains supplementary material available at 10.1186/s12885-022-09277-x.

## Background

Malignant pleural mesothelioma (MPM) is a rare thoracic malignancy that affects the pleura and is often associated with exposure to asbestos. Of newly diagnosed patients, the majority of patients present with an advanced disease are not suitable for surgery [1]. Despite the introduction of chemotherapy as the key treatment modality that has significantly improved survival, the median survival time of the patients is between 9 to 17 months [2]. Because the response rate to chemotherapy is only around 40%, refinements in the patient stratification have been sought [3].

CT is the standard radiological method used as an anatomical imaging method and to assess MPM response to treatment based on measuring the MPM thickness according to the modified response evaluation criteria in solid tumors (mRECIST) [4]. DCE-MRI is a functional imaging technique that has increasingly been implemented in conventional MRI protocols to assess intrinsic microvascular tumor properties. The quantitative analysis of DCE images enables the quantification of the blood supply to the tumors including the perfusion and permeability [5]. Among the calculated DCE parameters, *K*^trans^ (the volume transfer constant between the plasmatic and extravascular, extracellular space) and a semi-quantitative parameter iAUC (initial area under the gadolinium concentration curve) are supposed to be the main parameters that reflect the effect of chemotherapy [6]. The predictive value of pre-treatment DCE-MRI parameters as well as the early treatment induced change has been studied in malignant tumors at different locations [6, 7].

Thus far, one study has been conducted on MPM patients, however the researchers used the Brix model, which is the simplest quantitative model for the analysis that doesn’t allow the quantification of the *K*^trans^ parameter [8]. To overcome this shortcoming, we set out with a study where the DCE parameters are assessed by a commonly used model – the extended Tofts (ET), as well as a more complex model- the adiabatic approximation tissue homogeneity (AATH) model. Both models allow the assessment of more DCE-MRI parameters that provide additional information in the MPM tissue pathophysiology [9]. In our recent article, DCE parameters were correlated with chemotherapy response using mRECIST criteria, showing that high pre-treatment efflux rate constant between extravascular, extracellular space and plasma (k_ep_) values suggest better treatment response [10]. During the follow-up period, we obtained information on progression-free survival (PFS), overall survival (OS) recruiting also more patients.

The aim of the present study was to examine the survival predictive value of pre-treatment and early treatment induced changes of DCE parameters, with emphasis on *K*^trans^, in patients with MPM.

## Methods

### Patient population

We have prospectively included 32 consecutive patients with biopsy proven malignant pleural mesothelioma eligible for chemotherapy and treated at our institution from October 2013 until September 2015; 19 patients participated in a previous study [10]. The inclusion criteria for the study were as follows: all patients had to be older than 18 years of age, have histologically proven malignant pleural mesothelioma and Karnofsky performance status ≥60% or Eastern Cooperation Oncology Group performance status between 0 and 2. The exclusion criteria were as follows: other malignant disease (excluding in situ cervical cancer and non-melanocytic skin cancer), acute infection, other accompanying significant co-morbidities, peripheral sensory neuropathy grade ≥ 2 and vascular disorder grade ≥ 2 according to common terminology criteria (CTC) for adverse events 4.0., positive pregnancy test, absolute or relative contraindication to MRI and gadolinium administration.

Pre- and intra-treatment MR examination including DCE-MRI was scheduled for all 32 patients. Pre-treatment DCE-MRI was acquired in 28 patients (median time interval 10 days, range 0 - 24 days) before chemotherapy and in 4 patients before palliative intervantions as they rapidly clinically deteriorated and did not receive chemotherapy. Two patients died during the early part of chemotherapy, 1 patient was claustrophobic and refused further participation in the study and 2 patients had only a pre-treatment study as further MR acquisition was interrupted by technical difficulties. All remaining 23 patients had an intra-treatment study (median time interval 4 days, range 0-27 days). Nineteen patients received a first-line chemotherapy, 7 patients a second line chemotherapy and 2 patients a fourth line chemotherapy. After completing the chemotherapy, patients were followed-up every 2 months by the oncologist. The time point for analysis was August 2019.

Demographic and clinical data of the 32 patients is presented in Table 1. Data from individual patients is presented in Supplementary table A.1.

**Table 1:** Demographic and clinical data

	n (%)
No. of patients	32
Age (years), median (range)	67 (47-84)
PFS (median, IQR)	229 (130.5 – 480.5)
OS (median, IQR)	521 (161 – 708)
Gender (male)	25 (78.1%)
Histological type	
Epithelioid	24 (75%)
Sarcomatoid	2 (6.2%)
Biphasic	6 (18.7%)
Received line of chemotherapy during the study	
None	4 (12.5%)
1	19 (59.3%)
2	7 (21.8%)
4	2 (6.2%)
Treatment schemes	
Gemcitabine + cisplatin	14
Gemcitabine + cisplatin/carboplatin	3
Pemetrexed + cisplatin	7
Pemetrexed + cisplatin/carboplatin	4
Stage	
I	4 (12.5%)
II	2 (6.2%)
III	14 (43.7%)
IV	12 (37.5%)
Asbestos exposure	24 (75%)

*PFS* progression-free survival, *OS* overall survival, *IQR* interquartile range

### Survival assessment

Primary outcomes were OS, defined as the number of days from the first MR study to the death by any cause, and PFS defined as the number of days from the first MR study to the diagnosis of tumor progression during the treatment or in follow-up surveillance or death by any cause. Patients without progression or death at the time of the analysis were censored at the date of the close-out date for the data collection.

### Treatment

The treatment schema included gemcitabine and cisplatin [11, 12], or pemetrexed and cisplatin [13]. For patients with nephrotoxicity grade ≥ 2 and for those who reported nausea or vomiting grade 3 during the previous cycle according to CTC, cisplatin was replaced by carboplatin. No additional specific anticancer treatment was planned for patients in remission. Nevertheless, patients in remission with good performance status were again discussed at the thoracic tumor board for eventual surgery. Because of the heterogeneity of the clinical situation, there was no specific further line of a systemic therapy. As a general rule, patients who were previously treated with low dose gemcitabine and cisplatin, were treated with pemetrexed and either cisplatin or carboplatin or vice versa. Other treatment options included navelbine or palliative irradiation. Treatment was never prolonged at the expense of an unbearable quality of life.

### MR imaging protocol and analysis

MR images were acquired using a 3-T magnetic resonance system (Trio, Siemens Healthcare, Erlangen, Germany) with a 6- channel body matrix coil and phase array spine matrix coil in the supine position. The imaging protocol included respiratory triggered T2-weighted turbo spin echo sequence with fat saturation in axial plane (repetition time msec/echo time msec 3000/99; 24 sections with an 8-mm section thickness and 1.6 mm gap; field of view, 340 × 250 mm; matrix, 189 × 320; voxel resolution, 1.3 × 1.1 × 8 mm) and T1-weighted three-dimensional (3D) gradient-echo breath hold sequence (VIBE) before and after contrast agent administration (repetition time msec/echo time msec, 3.18/1.15; field of view, 346 × 324 mm; voxel size, 1.3 × 1.1 × 1.5 mm; matrix, 246 × 320; 96 slices, 1.5 mm slice thickness, 0:39 min imaging time) covering the whole thorax from clavicles to the diaphragm. DCE-MRI scans were performed over part of the thorax showing tumor burden using a T1-weighted tree-dimensional gradient echo sequence (turbo-FLASH) (repetition time msec/echo time msec, 4.5/1.16, flip angle, 15°, field of view, 330 × 330 mm; matrix, 192 × 192, voxel size, 1.7 × 1.7 × 5 mm, 30 slices per slab with a 5 mm section thickness, temporal resolution, 18 s per scan). Images were acquired during shallow breathing, a total of 20 sequential repetitions were acquired. Gadolinium contrast agent (Gadovist, Gadobutrol, Berlin, Germany) administration was done with after the third repetition at a dose of 0.1 mmol/kg followed by 30 ml of saline flush, both at the rate of 3.5 ml/s using a power injector (Medrad, Spectris Solaris EP). The T1 mapping was used to convert signal intensity into gadolinium concentration. T1 map was calculated from pre-contrast T1-weighted images acquired with 2 averages and flip angles of 2°, 10° and 15°.

The conventional and DCE-MR images were consensually reviewed by the two radiologists. The DCE images were transferred for post-processing to a separate workstation running commercially available software (Olea Medical 2.3, La Ciotat, France). Post-processing included motion correction and signal smoothening for converting signal intensities into a gadolinium concentration. Regions of interest (ROI) were drawn freehand around the MPM periphery on all axial post-contrast T1-weighted images avoiding large vessels, readily recognizable necrotic tissue (low-attenuation nonenhancing areas within tumors), adjacent atelectasis and surrounding normal tissue. ROIs were than propagated to all obtained axial DCE images. Contrast agent concentration calculation was performed as previously described [14]. Arterial input function was obtained by manually selecting the aorta. The software analysed transport processes by using two-compartment models: ET and AATH model, and provided quantitative DCE parameters representing the volume transfer constant *K*^trans^ (1/min), the plasma volume fraction v_p_ (ml/100 ml), extravascular extracellular volume fraction v_e_ (ml/100 ml), efflux rate constant k_ep_ (1/min), blood flow F (ml/min/100 ml), capillary transit time TC (min) and the extraction constant E (%), and the semi quantitative parameter representing the initial area under the gadolinium concentration curve iAUC (mM). Tumor TNM stage was determined according to the 7th edition of the TNM classification for MPM on the basis of results from chest MRI and PET-CT [15, 16].

### Statistical analysis

Data normality was tested in 315 876 tumor voxels in all patients (with a minimum number of values per parameter of 118 139 and a maximum number of 283 656) using the Kolmogorov-Smirnov test. As the DCE parameter values were non-normally distributed, non-parametric tests were used. Continuous variables are presented by the median values and the interquartile range (IQR). The change between pre- and intra-treatment values is expressed in percentage (%). Due to the linearly shaped PFS and OS curve, we performed the analysis of prognostic values of DCE parameters by dividing the patients into PFS and OS quartiles. A Mann-Whitney test for independent samples was performed for statistical testing the differences in pre- and intra-treatment DCE-MRI parameters as well as the change in DCE parameters in the first part of the treatment between patients having different histological types of MPM (epithelioid vs. sarcomatoid and biphasic) and disease stages (stage I and II vs. III and IV) and groups of patients in different PFS and OS quartiles (Q1 vs. Q2-4; Q1-2 vs. Q3-4; Q1-3 vs. Q4). The *P* value was adjusted for multiple testing (Bonferroni correction) and the value <.004 was considered statistically significant. Significant predictors for survival were identified using the univariate Firth’s bias-reduced logistic regression analysis. All variables that reached the level of statistical significance in univariate analysis were entered into the multivariate Firth’s bias-reduced logistic regression analysis to identify the independent predictors of PFS and OS. The receiver operating characteristics (ROC) curve analysis was applied to determine the discriminatory power of the DCE parameters. The area under the curve (AUC) was computed and the optimal cut-off values were calculated by selecting the highest Youden’s J statistic on the ROC curve, thereby maximizing sensitivity and specificity.

All data analyses and graphs were performed using MedCalc Statistical Software version 15.6.1 (MedCalc Software bvba, Ostend, Belgium; https://www.medcalc.org; 2015) and the R package logistf: Firth’s bias-reduced logistic regression (version 1.2.5001).

## Results

**Fig. 1:**
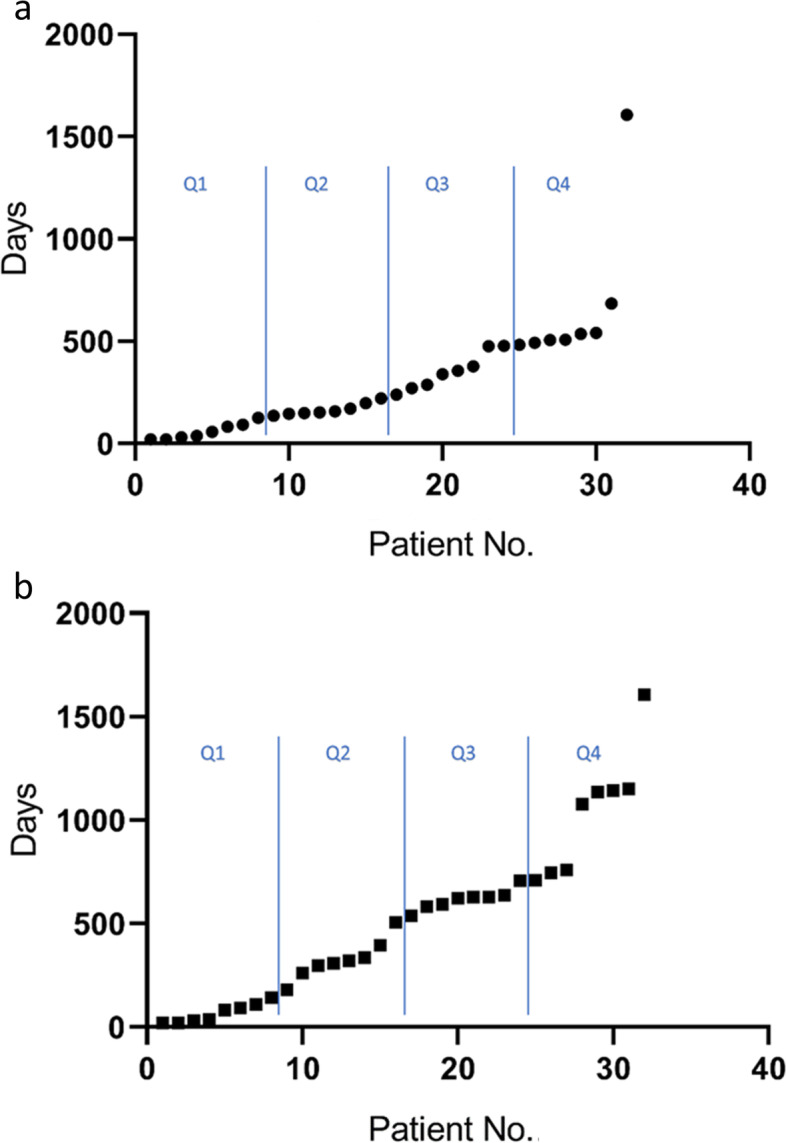
The progression free survival and overall survival of the patients (**a** and **b**). Q1, Q2, Q3 and Q4 indicate the first, second, third and fourth quartile, on both graphs. Both graphs are linearly shaped indicating that the number of patients with progression and patient deaths was stable over time

The median PFS was 229 days (7.5 months) (interquartile range = 130.5 – 480.5 days) and median OS was 521 days (17.1 month) (interquartile range = 161 – 708 days) as presented in Fig. 1. At the time of analysis, only 1 patient was alive.

Patients with epithelioid type MPM had significantly higher pre-treatment AATH-calculated *K*^trans^ compared to patients with sarcomatoid and biphasic type, 0.09 (0.8 – 0.11) min^-1^ vs. 0.05 (0.01-0.06) min^-1^, (*P* = .0008). Other DCE values didn’t significantly differ between patients having different histological types of MPM or disease stage.

Higher pre-treatment *K*^trans^ and v_e_ values calculated using both models were observed in patients showing significantly longer OS (Table 2). Specifically, ET- calculated *K*^trans^ values were 0.13 (0.09 – 0.20) min^-1^ vs. 0.07 (0.05 – 0.08) min^-1^ and AATH-calculated *K*^trans^ values were 0.11 (0.09 – 0.19) min^-1^ vs. 0.07 (0.05 – 0.09) min^-1^ in patients with OS>708 days compared to patients with OS<708 days. Also, ET- calculated v_e_ values were 34 (28 - 39) ml/100ml vs. 26 (17 – 30) ml/100ml in patients with OS>521 days compared to patients with OS<521 days and 35 (IQR 28 – 46) ml/100ml vs. 28 (22 – 35) ml/100ml in patients with OS>708 days compared to patients with OS<708 days. Patients with AATH-calculated v_e_ values 31 (24 – 46) ml/100ml had the lowest OS (<161 days) compared to patients with v_e_ values 44 (39 – 61) ml/100ml who has longer OS (>161 days).

**Table 2:** Comparison of DCE values and their changes according to the PFS and OS outcomes

Parameter	PFS > 130.5 days (Q2-4 > Q1)	PFS > 229 days (Q3-4 > Q1-2)	PFS > 480.5 days (Q4 > Q1- 3)	OS > 161 days (Q2-4 > Q1)	OS > 521 days (Q3-4 > Q1-2)	OS > 708 days (Q4 > Q1-3)
**Pre-treatment**						
ET-*K*^trans^	.17	.21	.06	.02	.02	**.008**
AATH-*K*^trans^	.27	.13	.26	.02	.02	**.008**
ET-k_ep_	.69	.69	.23	.13	.08	.003
AATH-k_ep_	.49	.17	.26	.08	.19	.05
ET-iAUC	.19	.89	.73	.02	.01	.23
AATH-iAUC	.26	.81	.79	.01	.01	.18
ET-v_p_	.98	.29	.72	.28	.36	.75
AATH-v_p_	.31	.33	55	.02	.04	.25
ET*-*v_e_	.09	.23	.21	.01	**.009**	**.007**
AATH-v_e_	.05	.07	.18	**.006**	.002	.04
TC	1	.55	.33	.44	1	.14
E	.81	.74	.05	.81	.24	.03
F	.32	.09	.98	.02	.05	.95
**Intra-treatment (between 3. and 4. cycle of chemotherapy)**						
ET-*K*^trans^	NA	.45	.36	NA	.06	.21
AATH-*K*^trans^	NA	.47	.11	NA	.05	.66
ET-k_ep_	NA	.41	.08	NA	.37	.95
AATH-k_ep_	NA	.63	.04	NA	.10	.95
ET-iAUC	NA	.87	.44	NA	.55	.33
AATH-iAUC	NA	.92	.40	NA	.59	.40
ET-v_p_	NA	.85	.29	NA	.39	.61
AATH-v_p_	NA	.53	.59	NA	.22	.66
ET*-*v_e_	NA	.09	.94	NA	.08	.06
AATH-v_e_	NA	.18	.48	NA	.14	.30
TC	NA	.39	.36	NA	.80	.57
E	NA	.92	.94	NA	.70	.21
F	NA	.77	.23	NA	.36	.57
**Change intra vs. pre-treatment studies**						
ET-*K*^trans^	NA	.73	**.008**	NA	.88	.16
AATH-*K*^trans^	NA	.97	.03	NA	.33	.29
ET-k_ep_	NA	.30	**.008**	NA	.47	.06
AATH-k_ep_	NA	.30	.11	NA	.33	.48
ET-iAUC	NA	.92	.26	NA	.17	.81
AATH-iAUC	NA	.92	.20	NA	.15	.12
ET-v_p_	NA	.73	.44	NA	.95	.95
AATH-v_p_	NA	.73	.73	NA	.75	.74
ET*-*v_e_	NA	.56	.22	NA	.56	.27
AATH-v_e_	NA	.18	.40	NA	.59	.57
TC	NA	.37	.67	NA	.56	.63
E	NA	1	1	NA	1	1
F	NA	.80	.35	NA	.62	.94

Units: *K*^*tran*s^ (1/min), k_ep_ (1/min), iAUC (mM), v_e_ (ml/100 ml),v_p_ (ml/100 ml), TC (min), F (ml/min/100 ml), E (%), PFS (days), OS (days), NA = not applicable due to the small number of patients in Q1 at this time point. Significant *P* values (<.004) are annotated in bold

Interestingly, a more prominent reduction in ET-calculated *K*^trans^ and k_ep_ values between intra- and pre-treatment study (-39% and -43%) was statistically significant for patients with longest PFS (>480.5 days) compared to patients with PFS<480.5 days (-9% and 20%), but showed no significance for OS.

To examine the predictive value of pre-and intra-treatment DCE parameters and their change for PFS and OS, we started by performed univariable Firth’s bias-reduced logistic regression analysis. (Supplementary table A.2, A.3 and A.4). Pre-treatment parameter values held no predictive value for PFS. Most parameters, with the exception of TC and E, were predictive for OS: ET and AATH-calculated *K*^*tran*s^, iAUC and v_e_ as well as AATH-calculated v_p_ were predictive for OS>161days; ET and AATH-calculated iAUC, ET-calculated *K*^*tran*s^, k_ep_ and v_e_, AATH-calculated v_p_ and F were predictive for OS>521 days and ET and AATH-calculated *K*^*tran*s^ and v_e_ were predictive for OS>708 days.

Intra-treatment values and early intra-treatment change were predictive for PFS: AATH-calculated k_ep_ was predictive for PFS>130 days while ET-calculated k_ep_ and *K*^*tran*s^ were predicative for PFS>480.5 days. Also, early intra-treatment change of AATH-calculated v_p_ was predicative for PFS>130.5 days, and AATH-calculated v_p_ and iAUC, ET-calculated k_ep_ and iAUC and F were predictive for OS>161 days.

Multivariable Firth’s bias-reduced logistic regression analysis demonstrated that only ET-calculated pretreatment *K*^*tran*s^ is an independent predictor for OS>708 days (*P* = .02) (Table 3). Other values have not reached a level of statistical significance as independent predictors of OS.

**Table 3:** DCE parameters as predictors of OS>708 days

	Log OR (95% CI)	OR (95% CI)	*P* value
ET- *K*^*tran*s^	65.51 (32.67 – 8.34)	2.8e+28 (4201 – 2.85e+78)	**.02**
AATH- *K*^*tran*s^	-21.33 (-90.95 – 17.76)	5.44e-10 (3.15e-40 – 5.17e+7)	.30
ET-v_e_	-15.75 (-68.30 – 11.68)	1.43e-7 (2.17e-30 – 1.18e+5)	.27
AATH- v_e_	6.05 (-14.62 – 43.46)	425 (4.45e-7 – 7.55e+18)	.27
(Intercept)	-2.71 (-6.88 – 0.43)	0.006 (0.001 – 1.54)	.08

Units: *K*^*tran*s^ (1/min), v_e_ (ml/100 ml), OS (days), *OR* odds ratio, *CI* confidence interval. Significant *P* value is annotated with bold

Epithelioid histological type was a favourable predicitve factor for OS>161 days (*P* =.008), OS>521 days (*P* = .0008) and OS>708 days (*P*. = .04), but it was not predictive for PFS. Disease stage held no predictive value for PFS or OS.

**Fig. 2:**
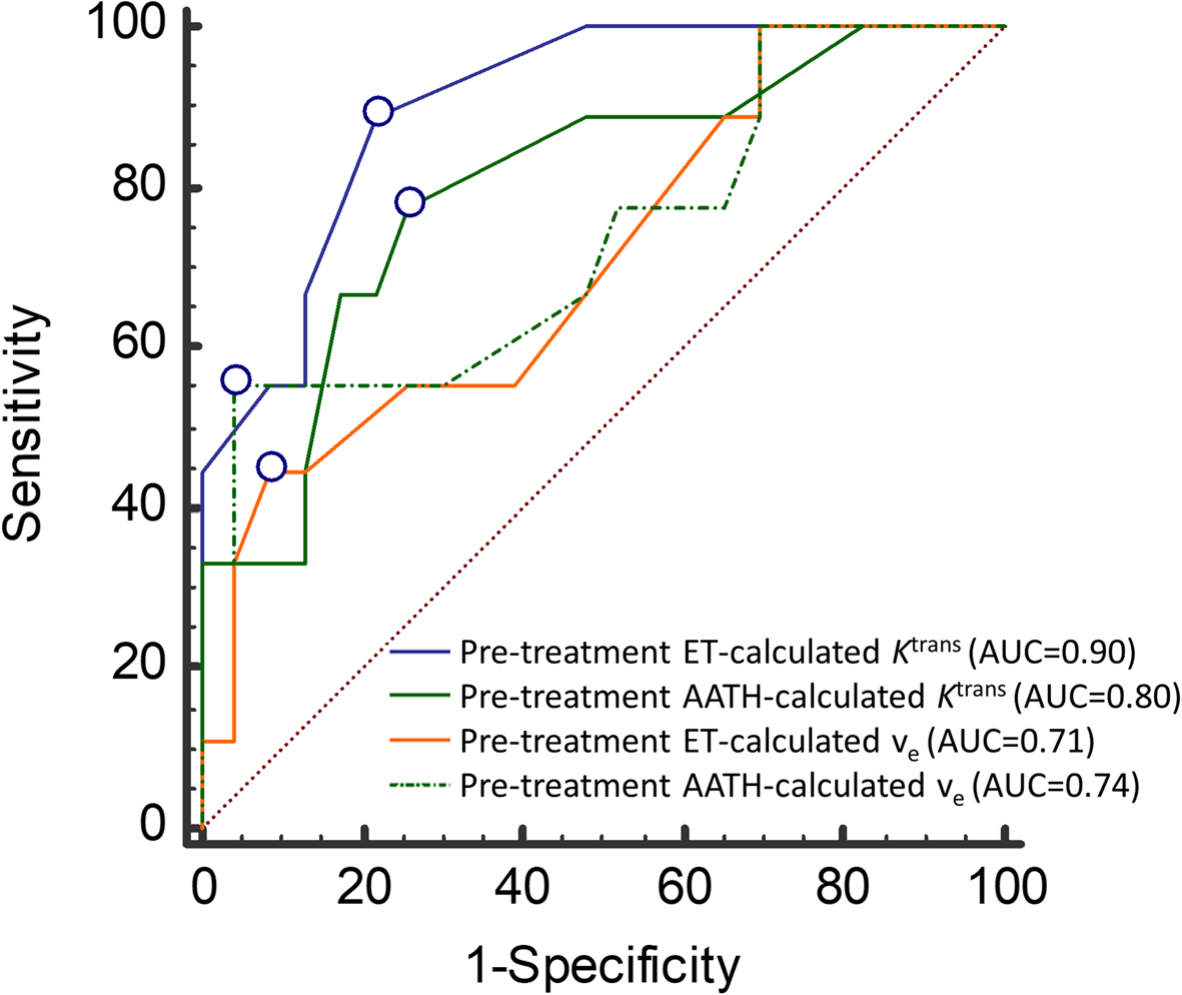
The ROC curves for predicting OS>708 days. The ROC curves for comparing discriminatory performances of pre-treatment ET and AATH-calculated *K*^trans^ an the v_e_ parameter values. The highest AUC was demonstrated by ET-calculated *K*^trans^ (AUC = 0.90). The circles indicate the Youden index

ROC curve analysis was used to identify DCE parameters that best discriminated patients with longer PFS and OS. DCE parameters that showed an excellent discriminatory performance for PFS>480.5 days were early intra-treatment changes in ET-calculated *K*^trans^ (estimated sensitivity/specificity, 83%/82%, *P* < .001, AUC = 0.87, 95% CI 0.66 – 0.97, criterion ≤ -14,29 min^-1^), AATH-calculated *K*^trans^ (estimated sensitivity/specificity, 100%/53%, *P =* .002, AUC = 0.81, 95% 0.59 – 0.94, criterion ≤ 0 min^-1^) and ET-calculated k_ep_ values (estimated sensitivity/specificity, 83%/88%, *P* < .001, AUC = 0.87, 95% CI 0.67 – 0.97, criterion -27,78 min^-1^). Pre-treatment AATH-calculated v_e_ was excellent for discriminating patients with OS > 161 days (estimated sensitivity/specificity, 75%/88%, *P* = .002, AUC = 0.83, 95% CI 0.65-0.94, criterion > 33 ml/100ml) and patients with OS > 521 days (estimated sensitivity/specificity, 87%/69%, *P* < .001, AUC = 0.81, 95% CI 0.63-0.92, criterion > 33 ml/100ml). The best discriminatory value of all DCE parameter was observed for pre-treatment ET-calculated *K*^trans^ values in patients with OS > 708 days (estimated sensitivity/specificity, 89%/78%, *P* < .001, AUC = 0.90, 95% CL 0.74-0.98, criterion > 0.08 min^-1^) (Fig. 2). Other DCE parameters achieved a weak to moderate discriminating performance.

**Fig. 3:**
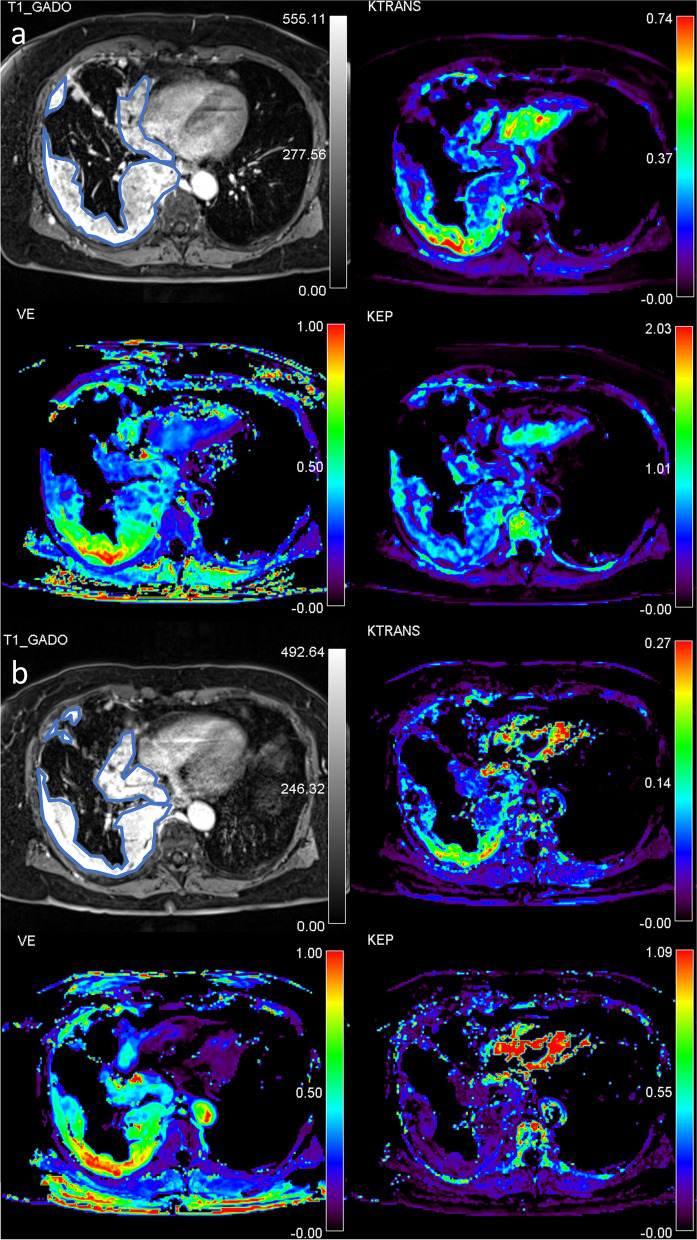
An example of a patient with long PFS and OS. Pre-treatment and intra-treatment DCE-MRI (**a** and **b**), a post-contrast T1 weighted-image is shown together with ET-calculated *K*^trans^, v_e_ and k_ep_ parametric maps. Regions of interest (ROI) are drawn around the MPM periphery on post-contrast T1 weighted-image. The pre-treatment median values were: *K*^trans^ = 0.22 min^-1^, v_e_ = 40 ml/100ml, and k_ep_ = 0.54 min^-1^ and the intra-treatment values were *K*^trans^ = 0.18 min^-1^, v_e_ = 48 ml/100ml, and k_ep_ = 0.39 min^-1^. The parametric maps show MPM spatial heterogeneity regarding its vascular properties

An example of pre- and intra-treatment DCE-MRI in patient with long PFS and OS is shown in Fig. 3.

## Discussion

Despite the advances in cancer treatment, the OS in patients with MPM remains unchanged since the introduction of pemetrexed in the treatment scheme [13]. The response to conventional cytotoxic chemotherapy is poor but varies substantially from patient to patient, even after taking into account the known prognostic factors such as histology, gender, stage and performance status [17]. DCE-MRI has the potential to impact therapeutic prognostication as it provides quantitative, non-invasive and longitudinal data on tumor vascular characteristics in individual patients. Cytotoxic chemotherapy is known to have a long term vascular disruptive effect which is why we chose to test DCE parameters as imaging biomarkers in MPM [18].

The findings of the study showed that pre-treatment *K*^trans^ was the strongest predictor of the tumor response to therapy and that the higher values result in a longer survival time. *K*^trans^ values reflect vessel wall permeability or blood flow, depending on the predominant effect. This result suggests that more permeable and/or highly perfused vasculature may provide better access to chemotherapy. *K*^trans^ could also reflect oxigenation and thereby predict the responce to radiotherapy [19]. Several studies that have proven that there is a correlation between the two transfer constants (*K*^trans^ and k_ep_) and survival [20]. Similar results with regard to prognostic significance of DCE parameters were obtained in patients with rectal cancer [21], breast cancer [22], osteosarcoma [23] and cervical cancer [24] treated with cytotoxic chemotherapy. All of the listed studies used the two-compartment models for DCE analysis. Contrary to our results, a study by Giesel et al. on MPM has demonstrated that lower pre-treatment k_ep_ values can be associated with better prognosis to cytotoxic chemotherapy [7]. Result comparison with this study is challenging, as Brix model for the DCE analysis was used, which is a simple quantitative model, doesn’t require T_10_ mapping and arterial input function measurement, and the effect of the peripheral compartment is assumed to be negligible to the central compartment. Hence, *K*^trans^ can’t be reliably assessed [25, 26]. Several studies have proven that there are significant differences in the obtained perfusion parameters when different models are used [25]. Model selection is important to correctly access the tumors vascular characteristics, determine the robust functional imaging parameter and to help establish their biomarker value. We used the extended Tofts model for DCE analysis which is the most widely used model and is suggested for drug development studies [25, 27]. Another model we used is the AATH model, which is a more elaborate model that allows separate calculation of flow and perfusion, all of which could be useful to clinicians. Interestingly, on univariate analysis, *K*^trans^ parameter was a significant predictor for OS regardless of the post-processing model, but on multivariate analysis only ET-calculated *K*^trans^ remained an independent predictor for OS. In our previous study, there was a small negative difference of -0.02 min^-1^ between the mean ET and AATH-calculated *K*^trans^ values, which were generally interchangeable between the models except in a MPM with the highest perfusion [10].

On the univariate analysis, the pre-treatment v_e_ also had a significant positive impact on OS. v_e_ represents the fractional volume of extracellular, extravascular space or the leakage space, where the contrast agent accumulates after diffusing from the capillaries. It has been found to coincide with areas of necrosis and apoptosis on histologic studies. A study on tumor xenografts showed that v_e_ highly negatively correlates with the intratumor cellularity [28, 29]. The precise physiological meaning of v_e_ is hard to interpret and its assessed value is highly dependent on other tracer kinetic parameters, primarily on capillary flow and permeability. In any case, higher v_e_ values reflect fast tumor kinetics, which is a known marker of malignancy [30, 31]. Higher pre-treatment *K*^trans^ and v_e_ values were found in patients with lung cancer showing responce to a combination of cytotoxic and anti-angiogenic therapy. Also, pre-treatment ET and AATH-calculated iAUC values were prognostic for OS in the univariate analysis. iAUC is regulated by permeability, blood flow, as well as wash out. It is resistant to poor fitting and is calculated from the area under the gadolinium uptake curve. It is an alternative end-point to *K*^trans^ in clinical trials as it can also identify highly perfused and permeable tumors but it lacks physiological interpretation [32].

Patients showing a 43% reduction in ET-calculated k_ep_ values and a 39% reduction in ET-calculated *K*^trans^ values intra-treatment experienced a significantly longer PFS. In a study by Giesel et al. responders also demonstrated a decrease in k_ep_ values, and non-responders demonstrated an increase in k_ep_ values during treatment [7]. Similarly, a reduction in *K*^trans^ and in k_ep_ values was observed in patients with osteosarcoma [23], while a reduction in *K*^trans^ values was observed in rectal cancer [21], breast cancer [22] and in colorectal liver metastasis [5]. It is difficult to establish, to which degree, the change in tumor vasculature is due to cytostatic and to which to anti-angiogenic vascular disruptive action. As we have evaluated the patients after 3 cycles of chemotherapy, a decrease in the two transfer constants can be due to tumor cell death and secondary blood flow or tumor vascular bed “(pseudo)-normalization” – pruning of ineffective vasculature and decrease in vascular permeability.

Lower intra-treatment ET and AATH-calculated k_ep_ values showed prognostic value for longer PFS on univariate logistic analysis but failed to confirm as an independent prognostic marker for OS. It is notable that the effect of anti-angiogenic treatment has been shown mostly on PFS, rather than OS [33]. Our results may therefore suggest an effect of cytotoxic therapy in MPM vasculature and an indication of the tumor adaptation the induction and upregulation of other pathways leading to tumor drug resistance. An evidence of a complex loop of resistance mechanisms offering opportunity for combinational treatment.

Our patients received standard chemotherapy including cisplatin with gemcitabine or pemetrexed. Patients with lower pre-treatment *K*^trans^ values – mostly with sarcomatoid and biphasic histology- were found to have a worse prognosis. If confirmed in larger trials, DCE-MRI could be used for patient stratification and identification of those who would benefit from other therapeutic options sparing them from less effective therapy as well as the consequent frequent adverse events that decrease the quality of life. These patients could be directed to clinical trials and could benefit from new approaches to treating mesothelioma such as mesothelin targeted therapy, anti-angiogenesis therapy or immunotherapy [34]. With the development of new treatment, that are targeted to a specific process, its effect in most patients may be small, but present and beneficial in a subgroup of patients [35]. Also, genetic and histological biomarkers that could help guide treatment selection have been identified but have not been used yet for treatment guidance [3].

The limitations of this study need to be acknowledged. The patient cohort is small, nevertheless, it is almost 2 times bigger than in the previous published study on the use of DCE-MRI to predict MPM patient outcome [7]. The optimal time point of evaluation is crucial for determining the effect of the therapy. We performed the measurement after 3 cycles of chemotherapy. A more frequent DCE measurement could provide helpful guideline for the follow-up timeline in clinical trial. Our patient cohort consisted of patients receiving different treatment schemes and lines of chemotherapy. Including only chemotherapy naïve patients could show a more pronounce change in DCE parameters during the chemotherapy. Furthermore, we included patients with all histologic types of MPM, of which epithelioid type is known to be assosiated with the most favourable prognosis [11]. A repeatability study, to assess the reproducibility of DCE parameters was not performed. Despite this, a reduction in *K*^trans^ values between 30-50% has been recognized to represent a significant change over the intrinsic variability [20]. We tried to perform a post-processing of DCE-MRI as standard as possible, but variations may arise due to the freehand region-of-interest delineation. Finally, despite the extended anatomic coverage in the DCE- MR scans, some tumour foci were not included in the study.

## Conclusions

In the present study, higher pre-treatment ET-calculated *K*^trans^ values were associated with longer OS. A decrease in ET-calculated k_ep_ values during the treatment and lower intra-treatment k_ep_ values were associated with longer PFS, which was not translated to longer OS. The findings suggest that there are variations in the MPM vascularity. It can be assumed that it could lead to a refocusing of therapeutic management from general to a more targeted approach in a subgroup of patients. Patients with lower pre-treatment *K*^trans^ values may benefit from other therapeutic options and patient with an intra-treatment decrease in k_ep_ values may benefit from a combination of therapies to impede early resistance pathways. Overall, the predictive ability of DCE-MRI biomarker is promising, and further validation should include prospective studies within the clinical trials.

## Supplementary Information

**Additional file 1: Table A.1.** Demographic and clinical data from individual patients.

**Additional file 2: Table A.2.** Analysis of pre-treatment DCE parameters for PFS and OS outcomes.

**Additional file 3: Table A.3.** Analysis of intra-treatment DCE parameters for PFS and OS outcomes.

**Additional file 4: Table A.4.** Analysis of change intra- vs. pre-treatment DCE parameters for PFS and OS outcomes.

## Data Availability

The datasets used and/or analysed during the current study are available from the corresponding author on reasonable request

## Abbreviations

AUC: Area under the curve
iAUC: Initial area under the gadolinium concentration curve
AATH: Adiabatic approximation tissue homogeneity
CTC: Common terminology criteria (CTC)
DCE-MRI: Dynamic contrast-enhanced MRI
DCE: Dynamic contrast enhanced
E: Extraction constant
ET: Extended Tofts
F: Blood flow
k_ep_: Efflux rate constant
*K*^trans^: Volume transfer constant between the plasmatic and extravascular, extracellular space
MPM: Malignant pleural mesothelioma
mRECIST: Modified response evaluation criteria in solid tumors
OS: Overall survival
PFS: Progression free survival
ROC: Receiver-operating characteristics
TC: Capillary transit time
v_p_: Plasma volume fraction
v_e_: Extravascular extracellular volume fraction
