# Supplementary material for: Dynamic contrast-enhanced MRI in malignant pleural mesothelioma: prediction of outcome based on DCE-MRI measurements in patients undergoing cytotoxic chemotherapy
Source: BMC Cancer. 2022 Feb 20;22:191. doi: 10.1186/s12885-022-09277-x (PMC8859879; doi:10.1186/s12885-022-09277-x)
Supplement: Supplementary file 1 — Additional file 1: Table A.1. Demographic and clinical data from individual patients. [file 12885_2022_9277_MOESM1_ESM.docx]

| Table A.1: Demographic and clinical data from individual patients | | | | | | | | |
| --- | --- | --- | --- | --- | --- | --- | --- | --- |
| Patient | Sex | Age (years) | Histology | Received line of chemotherapy during the study | TNM | Stage | PFS (days) | OS (days) |
| 1 | M | 72 | Epithelioid | 1 | T2N1M0 | IIIA | 684 | 759 |
| 2 | F | 71 | Biphasic | 1 | T3N2M0 | IIIB | 171 | 307 |
| 3 | M | 53 | Epithelioid | 2 | T1bN1M0 | IB | 492 | 592 |
| 4 | M | 62 | Epithelioid | 1 | T3N2M0 | IIIB | 149 | 638 |
| 5 | M | 73 | Epithelioid | 1 | T4N3M0 | IV | 220 | 707 |
| 6 | F | 76 | Epithelioid | 4 | T3N2M0 | IIIB | 535 | 1078 |
| 7 | F | 66 | Biphasic | None | T4N1M1 | IV | 30 | 30 |
| 8 | F | 75 | Biphasic | None | T4N3M0 | IV | 20 | 20 |
| 9 | M | 58 | Epithelioid | 1 | T3N0M0 | IIIB | 153 | 261 |
| 10 | M | 63 | Biphasic | 1 | T4N3M0 | IV | 136 | 142 |
| 11 | M | 77 | Epithelioid | 1 | T3N0M0 | IIIB | 483 | 746 |
| 12 | M | 76 | Epithelioid | 1 | T3N0M0 | IIIB | 476 | 709 |
| 13 | F | 47 | Biphasic | 1 | T3N2M0 | IIIB | 287 | 395 |
| 14 | M | 61 | Epithelioid | 1 | T1bN0M0 | IB | 239 | 320 |
| 15 | F | 47 | Biphasic | 2 | T4N0M0 | IV | 20 | 20 |
| 16 | M | 61 | Epithelioid | 1 | T2N2M0 | IIIA | 478 | 622 |
| 17 | M | 67 | Sarcomatoid | 2 | T4N0M0 | IV | 505 | 505 |
| 18 | M | 60 | Epithelioid | 1 | T1bN0M0 | IA | 339 | 1142 |
| 19 | M | 75 | Epithelioid | 1 | T3N0M0 | IIIB | 271 | 538 |
| 20 | M | 68 | Epithelioid | 2 | T3N0M0 | IIIB | 146 | 180 |
| 21 | M | 84 | Epithelioid | 1 | T2N0M0 | II | 157 | 582 |
| 22 | F | 67 | Sarcomatoid | 1 | T2N0M0 | II | 57 | 109 |
| 23 | M | 60 | Epithelioid | 1 | T3N0M0 | IIIB | 335 | 335 |
| 24 | M | 79 | Epithelioid | 2 | T4N0M0 | IV | 93 | 93 |
| 25 | M | 72 | Epithelioid | 2 | T4N1M0 | IV | 198 | 297 |
| 26 | M | 70 | Epithelioid | None | T3N1M0 | IIIB | 37 | 37 |
| 27 | M | 78 | Epithelioid | None | T4N0M0 | IV | 83 | 83 |
| 28 | M | 81 | Epithelioid | 1 | T4N1M0 | IV | 125 | 627 |
| 29 | M | 67 | Epithelioid | 4 | T4N0M0 | IV | 377 | 627 |
| 30 | M | 65 | Epithelioid | 2 | T1aN0M0 | IA | 1606 * | 1606 * |
| 31 | M | 50 | Epithelioid | 1 | T4N2M0 | IV | 507 | 1135 |
| 32 | M | 61 | Epithelioid | 1 | T3N0M0 | IIIB | 541 | 1151 |
| PFS, progression-free survival, OS, overall survival, * alive at the time point of the analysis | | | | | | | | |
