# Supplementary material for: Dynamic contrast-enhanced MRI in malignant pleural mesothelioma: prediction of outcome based on DCE-MRI measurements in patients undergoing cytotoxic chemotherapy
Source: BMC Cancer. 2022 Feb 20;22:191. doi: 10.1186/s12885-022-09277-x (PMC8859879; doi:10.1186/s12885-022-09277-x)
Supplement: Supplementary file 2 — Additional file 2: Table A.2. Analysis of pre-treatment DCE parameters for PFS and OS outcomes. [file 12885_2022_9277_MOESM2_ESM.docx]

| Table A.2: Univariate logistic regression analysis of pre-treatment DCE parameters for PFS and OS outcomes. | | | | | | | | | | | | |
| --- | --- | --- | --- | --- | --- | --- | --- | --- | --- | --- | --- | --- |
| **Parameter** | **PFS>130.5 days** | | **PFS>229 days** | | **PFS>480.5 days** | | **OS>161 days** | | **OS>521 days** | | **OS>708 days** | |
|  | OR (95%CI) | *P* value | OR (95%CI) | *P* value | OR (95%CI) | *P* value | OR (95%CI) | *P* value | OR (95%CI) | *P* value | OR(95%CI) | *P* value |
| ET-*K*^trans^ | 14076.30 (0.007 – 1.10e+15) | .26 | 9.65 (0.0005 – 1.12e+6) | .64 | 1192.16 (0.04 – 6.52e+8) | .17 | 1.92 (17.2 – 6.05e+27) | **.02** | 7.6 (219.45 – 1.73) | **.005** | 2.08 (2.83 – 7.01e+33) | **<.001** |
| AATH-*K*^trans^ | 1319.15 (0.003 – 1.09e+12) | .38 | 3032.75 (0.007 – 2.05e+10) | .23 | 30503.24 (0.03 – 1.99e+11) | .14 | 3.9e+9 (2.6 – 2.8e+23) | **.04** | 38164200 (8 – 1.20e+17) | .06 | 3.99 )389.7 – 4.7e+19) | **.005** |
| ET-k_ep_ | 7.57 (0.02 – 4852.16) | .52 | 7.74 (0.05 – 1930.79) | .42 | 57.33 (0.17 – 4.88e+4) | .17 | 352.95 (0.6 – 9.26e+5) | .07 | 323.32 (1.27 – 3591111) | **.04** | 1.4e+4 (50.2 -9.6e+10) | .1 |
| AATH-k_ep_ | 2.95 (0.003 – 6550.09) | .75 | 32.61 (0.08 – 37359.03) | .26 | 64.53 (0.08 – 105961) | .22 | 132.37 (0.08 – 1.69e+6) | .2 | 44.71 (0.10 – 58360.07) | .22 | 524.7 (0.65 – 1.9e+6) | .07 |
| ET-iAUC | 1.00 (0.99 – 1.00) | .16 | 1.00 (0.99 – 1.00) | .59 | 1.00 (0.99 – 1.00) | .94 | 1.0001 (1.00003 – 1.0002) | **.005** | 1.00008 (1.00002 – 1.0001) | **.005** | 1.00003 (0.99 – 1.0001) | .22 |
| AATH-iAUC | 1.00 (0.99 – 1.00) | .14 | 1.00 (0.99 – 1.00) | .53 | 1.00 (0.99 – 1.00) | .97 | 897.7 (2.4 – 4.27e+6) | **.02** | 1.00008 (1.00002 – 1.0001) | **.004** | 1.00003 (0.99 – 1.0001) | .22 |
| ET-v_p_ | 5.05 (3.57e-5 – 8.38e+6) | .79 | 24.21 (0.0006 – 3.22e+6) | .55 | 5.98 (1.54e-5 – 8.05e+5) | .76 | 10731.24 (0.01 -1.01e+12) | .2 | 1755.23 (0.03 – 1.43e+9) | .19 | 0.49 (9.04e-7 – 46866) | .9 |
| AATH-v_p_ | 5.94 (0.08 – 1270.36) | .43 | 3.36 (0.09 – 192.85) | .51 | 2.65 (0.04 – 161.38) | .63 | 7988.26 (9.91 – 1.91e+8) | **.005** | 149.79 (1.81 – 72003.48) | **.02** | 5.21 (0.09 – 344) | .4 |
| ET*-*v_e_ | 129.12 (0.10 – 1.33e+6) | .19 | 6.02 (0.02 -3161.66) | .53 | 17.49 (0.03 – 14684.22) | .36 | 1.31 (2.25 – 1.59e+9) | **.03** | 3313 (3.07 – 6.5e+7) | **.02** | 1.04 (1.46 – 7.42e+6) | **.04** |
| AATH-v_e_ | 265.54 (0.98 – 3.73e+5) | .05 | 27.51 (0.34 – 5635.43) | .14 | 27.13 (0.21 – 7345.89) | .18 | 7988.26 (9.91 – 1.19e+8) | **.005** | 372.82 (10.78 – 1.86e+7) | .19 | 932 (3.9 – 1.95e+6) | **.01** |
| TC | 0.99 (0.95 – 1.05) | .91 | 0.98 (0.93 – 1.02) | .43 | 1.02 (0.97 – 1.07) | .33 | 0.97 (0.93 – 1.02) | .33 | 1 (0.96 – 1.04) | 1 | 1.03 (0.99 – 1.08) | .12 |
| E | 7.11 (0.001 – 186782.97) | .66 | 2.04 (0.001 – 5030) | .85 | 874.54 (0.19 – 1.66e+7) | .11 | 7.11 (0.001 – 186782.9) | .66 | 129.33 (0.06 – 1.2e+06) | .21 | 9.92 (1.96e+2 – 5.4e+13) | .34 |
| F | 1.01 (0.98 – 1.04) | .36 | 1.01 (0.99 – 1.03) | .28 | 1.00 (0.97 – 1.02) | .85 | 1.04 (1.00 – 1.08) | **.01** | 1.02 (1.0006 – 1.05) | **.04** | 1.001 (0.97 -1.02) | .87 |
| Units: *K^tran^*^s^ (1/min), k_ep_ (1/min), iAUC (mM), v_e_ (ml/100 ml),v_p_ (ml/100 ml), TC (min), F (ml/min/100 ml), E (%), OR, Odds ratio; CI, 95% Confidence interval | | | | | | | | | | | | |
