# Supplementary material for: Dynamic contrast-enhanced MRI in malignant pleural mesothelioma: prediction of outcome based on DCE-MRI measurements in patients undergoing cytotoxic chemotherapy
Source: BMC Cancer. 2022 Feb 20;22:191. doi: 10.1186/s12885-022-09277-x (PMC8859879; doi:10.1186/s12885-022-09277-x)
Supplement: Supplementary file 3 — Additional file 3: Table A.3. Analysis of intra-treatment DCE parameters for PFS and OS outcomes. [file 12885_2022_9277_MOESM3_ESM.docx]

| Table A.3: Univariate logistic regression of intra-treatment DCE parameters for PFS and OS outcomes. | | | | | | | | | | | | |
| --- | --- | --- | --- | --- | --- | --- | --- | --- | --- | --- | --- | --- |
| **Parameter** | **PFS>130.5 days** | | **PFS>229 days** | | **PFS>480.5 days** | | **OS>161 days** | | **OS>521 days** | | **OS>708 days** | |
|  | OR (95%CI) | *P* value | OR (95%CI) | *P* value | OR (95%CI) | *P* value | OR (95%CI) | *P* value | OR (95%CI) | *P* value | OR(95%CI) | *P* value |
| ET-*K*^trans^ | 416.41 (1.53e-11 – 2.49e+15) | .65 | 126.93 (0.0001 – 3.6e+8) | .49 | 7.29e-5 (2.69e-12 – 304.41) | .22 | 9.96e+9 (0.003 – 4.45e+39) | .12 | 1.07 (0.44 – 9.85e+13) | .06 | 6254.55 (0.001 – 1.64e+12) | .27 |
| AATH-*K*^trans^ | 0.0009 (5.65e-21 – 1.75e+12) | .67 | 580.35 (3.87e-5 – 4.97e+10) | .45 | 1.03 (3.75e-21 . 6.22) | .08 | 2.62e+12 (1.19e-6 – 2.43e+54) | .23 | 3.74e+7 (0.55 – 7.19) | .06 | 7.97 (1.69e-7 – 4.59e+8) | .81 |
| ET-k_ep_ | 1.47e-3 (1.72e-11 – 938.47) | .37 | 0.05 (4.19e-5 – 17.22) | .31 | 0.0007 (5.92e-8 – 0.87) | **.04** | 13860.31 (0.05 – 1.44e+14) | .14 | 23.24 (0.07 – 21416.47) | .29 | 1.15 (0.002 – 714.54) | .96 |
| AATH-k_ep_ | 6.30 (3.04e-39 – 4.22e-2) | **.02** | 0.009 (6.13e-8 – 206.05) | .36 | 4.56e-7 (5.43e-15 – 0.36) | **.03** | 1.92e+7 (0.0005 – 1.2e+27) | .19 | 1.10e+4 (0.17 – 2.2e+10) | .10 | 1.11 (1.62e-5 – 51649.60) | .98 |
| ET-iAUC | 0.99 (0.99 – 1.00) | .95 | 0.99 (0.99 – 1.00) | .87 | 0.99 (0.99 – 1.00) | .16 | 1.00 (0.99 – 1.00) | .17 | 1.00 (0.99 – 1.00) | .42 | 0.05 (5.06e-7 – 1348.20) | .57 |
| AATH-iAUC | 1.00 (0.99 – 1.00) | .99 | 0.99 (0.99- 1.00) | .93 | 0.99 (0.99 – 1.00) | .14 | 1.00 (0.99 – 1.00) | .17 | 1.00 (0.99 – 1.00) | .40 | 0.92 (0.03 – 18.59) | .95 |
| ET-v_p_ | 3.52e-4 (2.74e-15 – 57908.90) | .38 | 3.00 (0.0002 – 76837.64) | .83 | 0.004 (4.91e-9 – 224.53) | .38 | 4.54 (3.44e-8 – 7.39e+13) | .87 | 77.82 (0.005 – 4.99e+6) | .38 | 0.05 (5.06e-7 – 1348.20) | .57 |
| AATH-v_p_ | 3.44 (0.01 – 1.42e+8) | .69 | 3.56 (0.19 – 112.99) | .40 | 0.24 (0.003 – 6.31) | .41 | 4.45 (0.02 – 1.32e+9) | .65 | 4.24 (0.22 – 146.89) | .34 | 0.92 (0.03 – 18.59) | .96 |
| ET*-*v_e_ | 756.75 (0.05 – 1.46e+10) | .19 | 29.82 (0.31 – 7323.16) | .15 | 0.45 (0.003 – 65.79) | .74 | 255.17 (0.14 – 1.00e+9) | .19 | 64.52 (0.59 – 23839.50) | .09 | 80.51 (0.48 – 5.16e+4) | .09 |
| AATH-v_e_ | 1.82 (0.004 – 2951.61) | .84 | 5.98 (0.31 – 192.59) | .23 | 0.17 (0.003 – 4.40) | .29 | 288.29 (0.02 – 9.23e+8) | .25 | 11.21 (0.54 – 502.50) | .12 | 3.19 (0.15 – 88.10) | .45 |
| TC | 1.00 (0.94 – 1.15) | .87 | 0.99 (0.95 – 1.03) | .71 | 0.02 (-0.01 – 0.07) | .19 | 0.97 (0.89 – 1.03) | .29 | 1.00 (0.96 -1.05) | .75 | 1.02 (0.98 – 1.06) | .33 |
| E | 0.15 (1.4e-9 – 4.5e+10) | .84 | 3.5 (0.0002 – 1.38e+5 | .79 | 1.10 (9.8e-8 – 44476.38) | .98 | 6.88e+6 (1.34e-5 – 1.52e+24) | .36 | 27.30 (0.002 – 2.42e+6) | .51 | 1102.2 (0.04 – 1.39e+8) | .18 |
| F | 0.99 (0.96 – 1.03) | .70 | 1.00 (0.99 – 1.03) | .38 | 0.99 (0.96- 1.01) | .44 | 1.00 (0.97 – 1.15) | .73 | 1.00 (0.99 – 1.02) | .39 | 0.99 (0.98 – 1.01) | .71 |
| Units: *K^tran^*^s^ (1/min), k_ep_ (1/min), iAUC (mM), v_e_ (ml/100 ml),v_p_ (ml/100 ml), TC (min), F (ml/min/100 ml), E (%), OR, Odds ratio; CI, 95% Confidence interval | | | | | | | | | | | | |
