# Supplementary material for: Dynamic contrast-enhanced MRI in malignant pleural mesothelioma: prediction of outcome based on DCE-MRI measurements in patients undergoing cytotoxic chemotherapy
Source: BMC Cancer. 2022 Feb 20;22:191. doi: 10.1186/s12885-022-09277-x (PMC8859879; doi:10.1186/s12885-022-09277-x)
Supplement: Supplementary file 4 — Additional file 4: Table A.4. Analysis of change intra- vs. pre-treatment DCE parameters for PFS and OS outcomes. [file 12885_2022_9277_MOESM4_ESM.docx]

| Table A.4: Univariate logistic regression analysis of change intra- vs. pre-treatment DCE parameters for PFS and OS outcomes. | | | | | | | | | | | | |
| --- | --- | --- | --- | --- | --- | --- | --- | --- | --- | --- | --- | --- |
| **Parameter** | **PFS>130.5 days** | | **PFS>229 days** | | **PFS>480.5 days** | | **OS>161 days** | | **OS>521 days** | | **OS>708 days** | |
|  | OR (95%CI) | *P* value | OR (95%CI) | *P* value | OR (95%CI) | *P* value | OR (95%CI) | *P* value | OR (95%CI) | *P* value | OR(95%CI) | *P* value |
| ET-*K*^trans^ | 1.95 (0.24 – 73.89) | .054 | 1.21 (0.43 – 3.54) | .71 | 0.13 (0.08 – 0.65) | **.008** | 1.46 (0.18 – 28.15) | .07 | 1.16 (0.41 – 3.38) | .77 | 0.44 (0.11 – 1.36) | .16 |
| AATH-*K*^trans^ | 1.47 (0.11 – 122.55) | .08 | 1.14 (0.30 – 4.55) | .84 | 0.11 (0.002 – 0.79) | .08 | 0.91 (0.065 – 27.23) | .09 | 1.91 (0.50 – 8.84) | .35 | 0.53 (0.09 – 2.16) | .38 |
| ET-k_ep_ | 0.75 (0.35 – 7.27) | .054 | 0.68 (0.21 – 1.27) | .23 | 0.01 (9.33e-6 – 0.59) | **.01** | 0.62 (0.28 – 1.69) | **.02** | 0.98 (0.52 – 2.06) | .95 | 0.30 (0.02 – 1.13) | .18 |
| AATH-k_ep_ | 0.21 (0.01 – 11.40) | **.03** | 0.34 (0.03 – 2.32) | .27 | 0.04 (0.00 – 1.02) | .052 | 0.83 (0.03 – 386.75) | .09 | 3.71 (0.47 – 78.52) | .23 | 0.48 (0.03 – 3.68) | .49 |
| ET-iAUC | 1.22 (0.01 – 68494.42) | .09 | 0.86 (0.07 – 11.64) | .90 | 0.34 (0.07 – 5.58) | .48 | 0.008 (1.56e-20 – 0.09) | **.005** | 0.16 (0.007 – 1.98) | .16 | 1.53 (0.10 – 20.33) | .74 |
| AATH-iAUC | 1.12 (0.05 – 87852.22) | .96 | 0.83 (0.07 – 10.92) | .88 | 0.32 (0.006 – 5.11) | .45 | 0.01 (7.49e-18 – 0.09) | **.004** | 0.16 (0.00 – 1.92) | .15 | 1.37 (0.09 – 17.30) | .81 |
| ET-v_p_ | 0.84 (0.33 – 7.98) | .07 | 1.03 (0.55 – 2.11) | .92 | 1.14 (0.54 – 2.12) | .69 | 0.52 (0.18 – 1.39) | **.01** | 1.10 (0.59 – 2.39) | .75 | 1.27 (0.66 – 2.59) | .44 |
| AATH-v_p_ | 2.77 (0.28 – 2.59e+33) | **.04** | 1.14 (0.39 – 3.57) | .81 | 0.68 (1.16 – 2.23) | .54 | 0.08 (4.59e-7 – 1.01) | **.006** | 0.89 (0.29 – 2.63) | .81 | 0.92 (0.27 – 2.81) | .88 |
| ET*-*v_e_ | 0.36 (0.004 – 40.48) | .61 | 1.26 (0.14 – 13.27) | .83 | 0.53 (0.03 – 5.89) | .62 | 0.15 (0.00 – 10.01) | .35 | 0.74 (0.07 – 6.93) | .78 | 0.26 (0.01 – 2.95) | .29 |
| AATH-v_e_ | 18.65 (0.29 – 16879.57) | **.02** | 2.02 (0.34 – 14.43) | .43 | 0.25 (0.02 – 1.85) | .18 | 1.08 (0.02 – 60.99) | .09 | 1.59 (0.27 – 10.60) | .60 | 1.35 (0.2 – 9.81) | .74 |
| TC | 0.89 (0.04 – 2474.55) | .09 | 1.64 (0.25 – 15.33) | .61 | 3.05 (0.40 – 24.72) | .27 | 0.89 (0.03 – 2467.96) | .09 | 1.63 (0.24 – 15.34) | .61 | 0.88 ( 0.09 – 6.21) | .89 |
| E | 0.77 (0.27 – 5.89) | .06 | 0.90 (0.45 – 1.84) | .76 | 0.54 (0.02 – 1.36) | .35 | 0.97 (0.34 – 841.40) | .09 | 1.00 (0.50 – 2.13) | .99 | 0.59 (0.07 – 1.36) | .25 |
| F | 1.11 (0.29 – 60.09) | .09 | 0.9 (0.45 – 2.20) | .95 | 0.97 (0.36 – 2.15) | .93 | 0.18 (0.01 – 0.08) | **.002** | 0.90 (0.41 – 1.98) | .79 | 1.05 (0.44 – 2.3) | .89 |
| Units: *K^tran^*^s^ (1/min), k_ep_ (1/min), iAUC (mM), v_e_ (ml/100 ml),v_p_ (ml/100 ml), TC (min), F (ml/min/100 ml), E (%), OR, Odds ratio; CI, 95% Confidence interval | | | | | | | | | | | | |
